# Supplementary material for: Spatial Variation in Stream-Water Bacterial Communities Across Sites Differing in Wild Chinese Giant Salamander Detection Frequency
Source: Biology (Basel). 2026 Sep 18;15(18):1647. doi: 10.3390/biology15181647 (PMC13604818; doi:10.3390/biology15181647)
Supplement: Supplementary file 1 [file biology-15-01647-s001.zip › Data Related to “Spatial Variation in Stream-Water Bacterial Communities across Sites Differing in Wild Chinese Giant Salamander Detection Frequency”/Microbial Data/result/04.AlphaDiversity/04.AlphaDiversity_ReadMe.pdf]

扩增子结果文件补充说明（04）

2023年 8月28 日

04.AlphaDiversity 【 alpha 多样性分析结果】

1 alpha_index_table 【alpha 多样性指数】

1.1 sample/group1/【样本及组 alpha多样性指数】

1.1.1 alpha_diversity_index.*.txt 【 alpha多样性指数】

Alph a - diversity 主要关注局域均匀生境下的物种 数目， 因 此也被称为生境内的多样性 。在分析中，选取 observed_features ，Chao1 ，Shannon ，Simpson ，good - coverage 几种不同的Alpha多样性指数 ，以表征样品中物种分布的多样性和均匀度 ，并直观展示测序深度和数据 量情况。

列标题	含义
Sample/ group	样本/分组
observed_features	直 观观测到的物种数目（也即是 ASV 数目 ）
Shannon	样品中的分类总数及其占比。群落多样性越高 ，物种分布越均匀 ，Shannon 指数越大。
Simpson	表 征群落 内物种分布 的多样性和均匀度
Chao1	估计群落样品中包含的物种总数
dominance	它表示随机选取两条序列属于不同分类（如OTUs）的概率
goods_coverage	测序深度指数
Pielou_e	衡量物种个体数分布的均匀程度

simpson 有 3 种展示形式 ， 即 Simpson's Index (D) ，Simpson's Index ofDiversity (1 - D) 和 Simpson's Reciprocal Index (1 / D) ，它们对于反映群落多样性的效果相 近但是计算的结果形式不同；我们用的是 Simpson's Index ofDiversity (1 - D) 。

2visual_specaccum 【物种累积曲线】

2.1 sample 【样本的物种累积曲线】

2.1.1specaccum_test.{pdf,png} 【样本的物种累积曲线图】

3 visual_rankAbundance 【Rank Abundance 曲线】

3.1 sample/group1【样本及组 Rank Abundance 曲线】

3.1.1 rank_abundance.{sample,group}.{pdf,png}【 Rank Abundance 曲线展示】

4 visual_rarefaction【稀释曲线】

4.1 sample/group1【样本及组稀释曲线】

4.1.1 */*.{sample,group}.{pdf,png,xls}【(chao1, goods_coverage, observed_species, shannon, simpson, PD_whole_tree)稀释曲线展示】

5.visual_alphaDiversity 【组间 Alpha 多样性比较箱型图】

5. 1 * 【组间(dominance,chao1,goods_coverage,observed_features,pielou_e,shannon,simpson)比较箱型图】

5. 1. 1 *. {png,pdf} 【 Alpha 多样性指数箱型图】

5.1.2 *_test. {txt,pdf,png} 【组间T-test检验及可视化（组内样本数>2， 组数=2）】

列数	列标题	含义
1	Group_Pair	两两分组
2	p_value	P 值

5.1.3 *_two_wilcox. {txt,pdf,png}【组间wilcox秩和检验及可视化（组内样本数>2，组数=2）】

列数	列标题	含义
1	Group_Pair	两两分组
2	p_value	P 值

5.1.4 *_Tukey.{txt,pdf,png}【多组间方差 Tukey分析及可视化（组内样本>2，组>2)】

列数	列标题	含义
1		两两分组
2	diff	均值差
3	lwr	置信区间下限
4	upr	置信区间上限
5	p adj	P 值

5.1.5 *_kruskalWallis.txt. {txt,pdf,png}【多组间方差 Tukey分析及可视化（组内样本>2，组>2)】

列数	列标题	含义
1		两两分组
2	Difference	组间秩次差
3	pvalue	P 值
4	sig.	是否显著，若 p 值<0 .05，标*，p 值<0 .01 标 ***
5	LCL	置信区间下限
6	UCL	置信区间上限
